# Perceived Associations between Excessive Sugar Intake and Health Conditions

**DOI:** 10.3390/nu14030640

**Published:** 2022-02-02

**Authors:** Marília Prada, Magda Saraiva, Margarida V. Garrido, Ana Sério, Ana Teixeira, Diniz Lopes, Diana A. Silva, David L. Rodrigues

**Affiliations:** 1 Department of Social and Organizational Psychology, Iscte-Instituto Universitário de Lisboa, CIS_Iscte, Av. das Forças Armadas, Office AA110, 1649-026 Lisboa, Portugal; magda.saraiva@iscte-iul.pt (M.S.); margarida.garrido@iscte-iul.pt (M.V.G.); anasofiaserio@gmail.com (A.S.); amft31876@gmail.com (A.T.); diniz.lopes@iscte-iul.pt (D.L.); dflrs@iscte-iul.pt (D.L.R.); 2 Departamento Médico do Clube de Futebol “Os Belenenses”, 1449-015 Lisboa, Portugal; d_andrade_silva@hotmail.com

**Keywords:** free sugars, excessive sugar intake, health conditions, non-communicable diseases, diseases prevention

## Abstract

Excessive sugar intake represents an increased risk of developing non-communicable diseases (e.g., obesity, cardiometabolic diseases, and dental diseases). Still, it is unclear whether people are aware of these adverse health outcomes. The current study systematically examined the extent to which people associate health conditions with excessive sugar intake. Participants (*N* = 1010 Portuguese volunteers) freely reported all health conditions they associated with excessive sugar consumption and rated the strength of these associations for eight specific health conditions. All participants reported health conditions associated with excessive sugar intake, with the most frequent being risk factors for cardiometabolic diseases (e.g., diabetes), cardiovascular diseases, oral problems, oncological and mental health conditions. Moreover, participants considered diabetes, overweight/obesity, and oral problems as being the conditions most related to excessive sugar intake. Women, participants with children in the household, and experts in health/nutrition rated excessive sugar intake as being more strongly linked to some of the health conditions. The identification of the health conditions that people associate with excessive sugar consumption may inform policymakers, educators, and health professionals and support interventions targeting the general public or specific groups (e.g., overweight people) in raising awareness of potential adverse health outcomes and, ultimately, contribute to reducing sugar intake.

## 1. Introduction

Poor eating habits, including high sugar intake, contribute to a decrease in the average life expectancy and are associated with many health conditions [1,2]. The excessive intake of free sugars increases the overall energy intake and may reduce the intake of nutritionally richer foods, and it has been associated with multiple adverse health outcomes (for a review, see [3]). 

Free sugar is ubiquitous in current diets, corresponding to “all monosaccharides and disaccharides added to foods by the manufacturer, cook or consumer, plus sugars naturally present in honey, syrups and fruit juice concentrates” [2]. Its excessive intake has been associated with an increased risk of developing non-communicable diseases, such as overweight or obesity [4], cardiometabolic diseases [5], elevated blood pressure [6], some types of cancer [7,8], and dental caries [9], among others. For example, excessive sugar intake can predispose individuals to increased adiposity and, ultimately, overweight and obesity [10,11], especially when this consumption co-occurs with low levels of physical activity. Obesity, in turn, is currently considered a global epidemic with severe individual and societal implications, being also a risk factor for multiple health conditions [12]. In Portugal, the prevalence of obesity is particularly alarming (e.g., 28.6% of the individuals aged between 25 and 74 years are obese [13]). 

Research has also suggested a relationship between excessive sugar intake and cardiometabolic diseases, which includes both cardiovascular disease and conditions such as metabolic syndrome and type 2 diabetes. The common pathogenic mechanisms include inflammation, insulin resistance, lipid accumulation, and increased oxidation [14,15]. To illustrate, the increase in hypertension prevalence is mainly related to the aging of the population and exposure to various lifestyle risk factors, such as unhealthy diets [16]. In 2010, estimates suggested that 31.1% of adults (1.39 billion people) worldwide were hypertensive [16]. In Portugal, this cardiovascular disease affects 36% of the population aged between 25 and 74 years, and 71% of the population aged between 65 and 74 years [17]. Furthermore, diabetes affects 9.8% and 23.8% of the individuals in these age groups [18]. Another negative health outcome associated with excessive sugar consumption is dental diseases (e.g., dental caries and erosion [19]). It is estimated that more than 80% of the world’s population is affected by this disease [20], including Portugal (e.g., oral-health problems affecting 69–90% of Portuguese school-age children [21]).

Based on the evidence linking sugar consumption and adverse health outcomes, the WHO [2] has recommended limiting free-sugars intake to below 10% (ideally below 5%) of the daily total energy intake, which corresponds to 50 g/day for an average adult. Although several countries have implemented a set of strategies and measures to reduce sugar intake (for a review, see [22]), the available data show that this goal has yet to be met. For example, a recent report [23] showed that, in Portugal, the mean intake of sugars (mono and disaccharides) is 84 g/day (i.e., 18.5% of the daily total energy intake), whereas the mean intake of free sugars is 35 g/day (i.e., 7.5% of the daily total energy intake). The contribution of free sugars for the daily total energy intake is higher for adolescents and children (i.e., 10.5% and 9.6%, respectively). The most significant contributors to these consumption patterns are table sugar, sweets (e.g., candy, chocolate, and ice cream), and soft drinks. Critically, 24.3% of the general Portuguese population, 48.7% of the adolescents, and 40.7% of the children exceed the WHO’s recommendations regarding free-sugars intake [23].

Considering these consumption patterns, it becomes highly relevant to examine consumers’ perceptions regarding sugar intake and the expected outcomes of excessive consumption. Qualitative studies provide good insights into this topic. For example, a recent study using focus groups with a sample of 40 Portuguese college students showed that, overall, participants shared the view that sugar intake is essential for the body’s normal functioning and not harmful if eaten in moderation [24]. This study also showed ambivalence toward sugar intake, with participants expressing their love for sweets while characterizing sugar as something highly negative, processed, and addictive. Regarding the consequences of excessive sugar intake, participants often mentioned obesity, diabetes, cardiovascular diseases, skin problems, and general conditions (e.g., sugar as a cause for inflammation in different parts of the body). In another recent study, including 42 interviews with Portuguese parents, children’s excessive sugar intake was also associated with diverse conditions (e.g., overweight and obesity, diabetes, oral health problems, and cardiovascular and oncological diseases), including agitation, inattention, and hyperactivity [25] (see also [26] for similar results with a sample of Australian parents). The fact that people frequently associate excessive sugar intake with behavioral problems, such as hyperactivity disorder, is of particular interest, given the lack of supporting scientific evidence (for a meta-analysis, see [27]). Still, these studies have also suggested that individuals may be unaware of the mechanisms by which sugary products contribute negatively to their health [26] or downplay the impact of the adverse consequences of excessive sugar intake. For example, some people express the belief that sugar has a less negative impact than other nutrients or substances found in food, such as fat or preservatives, or express mistrust in information conveying the association between sugar intake and health conditions [24]. In other cases, individuals acknowledge the negative health outcomes but downplay the likelihood of experiencing them in the near future [24,28].

To the best of our knowledge, studies that are focused on examining the perceived health consequences of excessive sugar intake are still scarce. Although the reviewed qualitative studies provide important cues in this regard, these findings cannot be generalized, because they are based on small and specific samples (e.g., see [24,25]). Still, a few studies about sugar-sweetened beverages (SSB) consumption tackled this issue. For instance, research with large samples of US adults has shown that participants often associated weight gain and diabetes with excessive SSBs consumption [29,30]. However, because both studies required participants to select health conditions from a predefined set, this method does not allow to infer whether participants spontaneously identified these conditions, nor if they would also report other health conditions. In contrast, a recent study with Australian adults [28] included an open-ended question about the health effects associated with SSB consumption, and type 2 diabetes, weight gain, and heart disease were the most frequent responses. It is noteworthy that all these studies focused on conditions resulting from drinking SSBs (and not from overall excessive sugar consumption). It is possible that the associations with these negative health outcomes are driven from the unhealthfulness appraisal of SSBs, which, in turn, may result from several perceived attributes and not exclusively by their high sugar content (e.g., presence of additives, artificial sweeteners, caffeine content, fruit, etc. (see [31,32]). Moreover, these studies do not inform about the strength of the association between sugar intake and a given health condition.

Understanding the anticipated consequences of excessive sugar intake is relevant to developing strategies to address this problem. Hence, in the current study, we systematically examined Portuguese individuals’ perceptions about the health-related consequences of high sugar intake by using both a free-association task and a rating task, in which participants rated the strength of the association between excessive sugar intake and a set of eight health conditions (e.g., diabetes, cancer, and hyperactivity). Additionally, we explored if these ratings varied according to sociodemographic variables (e.g., gender, age, and education level; see also [28]) and correlated with other self-reported measures (e.g., frequency of high-sugar foods intake and attention to sugar-content information).

## 2. Method

### 2.1. Participants

This study included a sample of 1010 Portuguese volunteers (76.7% women, 22.9% men, and 0.4% other), aged between 18 and 82 years (*M* = 36.33, *SD* = 13.22). Half of the sample cohabited with a partner (50.0%), and most had no children in their household (66.5%). Most participants were employed (77.5%) and had a college degree (78.7%). Most participants reported following an omnivorous diet (72.5%) and were within the normal weight range (59.0%; 18.5 < BMI < 25). Still, 30.9% were overweight or obese (BMI > 25), 4.3% were underweight (BMI < 18.5), and 5.8% chose not to disclose this information. Moreover, only 12.6% reported having at least one diagnosed health condition that restricts their eating behavior. The most frequent health conditions were food allergies or intolerances (43.3%), pre-diabetes or diabetes (11.8%), and high cholesterol (10.2%). 

### 2.2. Procedure and Instruments

This study was part of a broader project about eating behavior (for other instruments and results, see [22,33]), and we only focus on the relevant measures for the current paper. All procedures were reviewed and approved by an ethics committee of the Iscte-Instituto Universitário de Lisboa (approval #22/2019).), and all methods were performed by following the ethical guidelines and regulations of the host institution. Written informed consent was obtained from all participants. Instructions stated the goals and the duration of the study, as well as ethical considerations (e.g., anonymity, confidentiality, and possibility to withdraw at any time). The invitation to participate in a web survey (hosted in Qualtrics) about eating habits was shared via mailing lists and on social networks (e.g., Facebook and LinkedIn). Data were collected from 7 February to 19 February 2019, and all Portuguese adults were eligible to participate. The only incentive to participate was the opportunity to enter a raffle (three 50€ gift cards). The contact information provided to enter the raffle was archived in a separate database and subsequently deleted.

The goal of the main task was to understand the perceived health outcomes of sugar intake. First, participants were asked whether they associated excessive sugar intake with any health condition (1 = *Yes*, 2 = *No*). Those who responded affirmatively were asked to write all the health problems they could remember. Next, we presented a list of eight conditions (in random order) that have been associated with high-sugar diets—hypertension, diabetes, overweight/obesity, dental caries or other oral health problems, cancer, high cholesterol, kidney disease [2,3], and hyperactivity. Participants were asked to indicate to what extent they considered each condition to be associated with excessive sugar intake (1 = *Not at all associated* to 7 = *Strongly associated*). Table 1 presents the measures used to assess participants’ perceptions or behaviors toward sugar.

Participants also answered sociodemographic questions (e.g., gender, age, occupation, and education level) and questions about their overall lifestyle and health status (e.g., weight/height; type of diet; and the following two questions, “In general, you would say that your lifestyle is...”, 1 = *Very sedentary* to 7 = *Very active*, and “In general, you would say that your health status is...”, 1 = *Very bad* to 7 = *Very good*). At the end of the survey, participants were thanked and fully debriefed. 

### 2.3. Data Analytic Plan

A total of 1483 participants agreed to participate in the survey. Only completed surveys were included in our analyses (*n* = 1010, 68.11% completion rate). A sensitivity power analysis using G*Power [36] indicated that the sample size allowed enough power (95%) to detect a small effect size of f(V) = 0.15. Data were analyzed with SPSS v29, and significance levels for inferential analyses were set to 0.05. 

Our primary analyses included the categorization of participants’ responses to the open-ended question about the health conditions they spontaneously associate with excessive sugar intake. We present the frequency observed for each health-condition category (Section 3.1). Moreover, a repeated-measures ANOVA was used to examine if the eight health conditions differ regarding how strongly they are associated with excessive sugar (multiple comparisons between health conditions with Bonferroni correction, Section 3.2). As additional analyses, we used independent-samples *t*-tests to explore if the perceived association between excessive sugar intake and each health condition varied according to individual characteristics (i.e., gender, presence of children, and expertise in health and nutrition). Finally, in Section 3.3, we use Pearson’s Correlation Coefficient to explore the pattern of associations between the overall association with health conditions and the variables related to the perception and behavior toward sugar and participants’ reported lifestyle.

## 3. Results

### 3.1. Excessive Sugar Intake and Health Conditions: Spontaneous Associations

**Figure 1:**
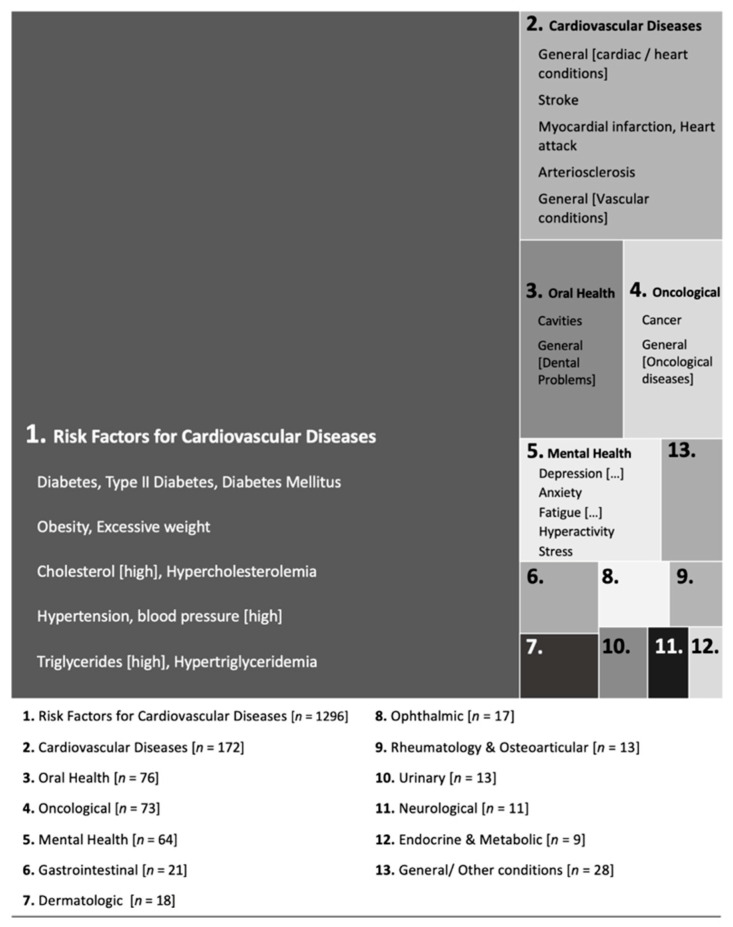
Categories of health conditions associated with excessive sugar intake. Note. The total number of health conditions = 1812. The area of each response category corresponds to its absolute frequency. Examples of health conditions are provided for the five most frequent categories.

The results indicated that only 13.7% (*n* = 138) of participants did not report any association between excessive sugar intake and health conditions. The remaining 86.3% (*n* = 872) responded “yes”, but 76 participants did not further indicate any health condition(s). In total, 796 participants indicated at least one health condition associated with excessive sugar intake (total of 1812 responses). These responses were categorized by two expert judges (one nurse and one general practitioner [37,38]). Different designations for the same health condition (e.g., “cholesterol”/“hypercholesterolemia”/“high cholesterol”) were aggregated. Results are summarized in Figure 1, and Table 2 presents the health conditions frequencies per category.

As shown in Figure 1, most participants associated excessive sugar intake with conditions that constitute risk factors for cardiovascular diseases. Other health conditions categories (although much less pervasive) were cardiovascular diseases, oral health problems, oncological diseases, and mental-health conditions.

Within risk factors for cardiovascular diseases, diabetes and obesity/overweight were widespread associations (56.1 and 32.3% of the total number of responses). Indeed, these health conditions were mentioned by 72.0 and 41.5% of the total sample, respectively. Cardiovascular diseases were primarily described in general terms (e.g., “heart conditions”). For the oral-health category, the most frequent associations were cavities. In the case of oncological diseases, most responses were specifically “cancer”. The mental-health category was more heterogeneous and included responses such as depression, anxiety, or fatigue. Noteworthy, the remaining responses (i.e., categories 6 to 13) were mentioned by very few participants (less than 1% of the total sample) and included diseases associated with diverse systems/organs (e.g., gastrointestinal, dermatologic, ophthalmic, etc.).

### 3.2. Excessive Sugar Intake and Health Conditions: Ratings of Strength of Association

**Figure 2:**
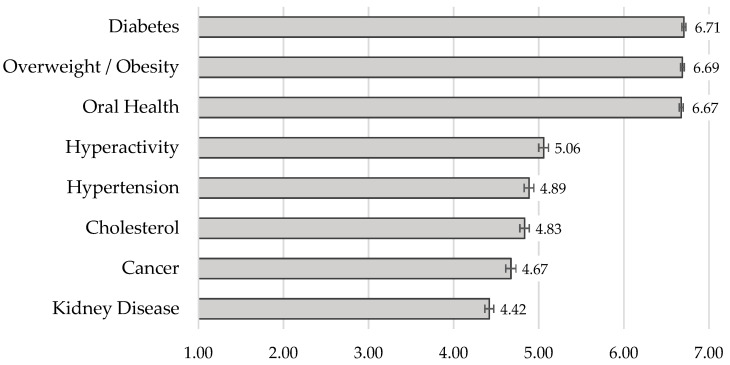
Mean ratings of perceived association between excessive sugar intake and a set of health conditions. *Note*. Error bars represent standard errors. Higher ratings reflect a perceived stronger association between excessive sugar intake and a given health condition (maximum = 7).

A one-way ANOVA with repeated measures showed that the strength of the association varied according to the health condition, *F*(5.04, 5089.23) = 582.54, *MSE* = 1422.98, *p* < 0.001, η_p_^2^ = 0.366 (with Huynh–Feldt correction as sphericity assumption was not verified). Overall, participants perceived all health conditions to be associated with sugar consumption (*M* = 5.49, *SD* = 0.85). As shown in Figure 2, diabetes, overweight/obesity, and oral-health problems were perceived as having the strongest associations with excessive sugar intake (vs. all other health conditions, all *p* < 0.001; post hoc tests with Bonferroni correction). Kidney disease obtained the lowest association ratings (vs. all other health conditions, all *p* ≤ 0.001). 

We also explored if the perceived association between excessive sugar intake and each health condition varied according to individual characteristics (see Table 3). Women (vs. men) rated excessive sugar intake as more associated with overweight/obesity, *t*(349.06) = 2.71, *p* = 0.007, *d* = 0.69; oral health, *t*(354.60) = 2.43, *p* = 0.016, *d* = 0.73; cancer, *t*(1004) = 5.94, *p* < 0.001, *d* = 1.91; and hyperactivity, *t*(1004) = 3.21, *p* = 0.001, *d* = 1.85. Moreover, we found that participants with (vs. without) children perceived a stronger association between sugar and cancer, *t*(1008) = 3.15, *p* = 0.002, *d* = 1.93; and hyperactivity, *t*(1008) = 3.03, *p* = 0.002 *d* = 1.85. Experts (vs. non-experts) provided higher ratings for the association between sugar intake and diabetes, *t*(199.46) = 2.27, *p* = 0.024, *d* = 0.72; cancer, *t*(167.11) = 6.47, *p* < 0.001, *d* = 1.86; obesity, *t*(175.92) = 2.46, *p* = 0.015, *d* = 0.64; and kidney disease, *t*(793) = 3.26, *p* = 0.001, *d* = 1.63. 

In contrast, we did not observe differences in the ratings for any of the health conditions according to BMI level (i.e., normal weight vs. overweight), all *p* > 0.203, or education level (i.e., with vs. without higher education), all *p* > 0.111, except for kidney disease, such that participants with higher education (*M* = 4.48; *SD* = 1.64) perceived a stronger association between this condition and excessive sugar intake than participants without higher education (*M* = 4.20; *SD* = 1.83), *t*(1008) = 2.20, *p* = 0.028. 

### 3.3. Correlations

As shown in Table 4, participants who perceived the health conditions as more associated with sugar intake also reported higher intention to reduce sugar intake, to attend to information about sugar more frequently, and to perceive more health benefits from reducing their sugar intake, as well as considering more critically the reduction of sugar intake in Portugal, all *p* < 0.001. Importantly, higher associations with health conditions were also negatively associated with the frequency of sugar intake, *p* = 0.006. Still, these associations were very weak in magnitude.

## 4. Discussion

The primary goal of this study was to examine the health conditions that participants associated with excessive sugar intake. We achieved this by asking participants to describe all health conditions they associated with excessive sugar intake and rate this association’s strength for eight conditions. We also explored whether individual characteristics determined these association ratings.

Overall, we observed that most participants reported at least one health condition associated with excessive sugar intake. The most frequent association was related to risk factors for cardiovascular diseases, such as diabetes and overweight/obesity, followed by actual cardiovascular diseases. Although much less prevalent than these two categories, participants also mentioned the association with cavities, cancer, and conditions related to mental health. The remaining responses were relatively infrequent but allowed us to characterize the spectrum of associations to excessive sugar intake. For instance, participants mentioned conditions affecting specific organs (e.g., the skin, eyes, bones, joints, etc.) and general adverse health outcomes (e.g., sugar as contributing to inflammatory processes or a weakened immune system). We also chose to include examples of the terms or expressions mentioned by participants that may not correspond to the medical designations (e.g., “heart attack” used as equivalent to “myocardial infarction”). This may provide important cues to health professionals about how to communicate about specific conditions with their patients. According to a recent study [39], the use of technical terminology, medical vernacular, acronyms, and abbreviations is still common in certain clinical contexts. To empower patients to manage their conditions, doctors are advised to consider their patients’ level of health literacy when communicating, using lay terms, and avoiding the use of medical jargon whenever possible [40].

The results of the open-ended question are in line with those of the rating task, in which diabetes, overweight/obesity, and oral-health problems emerged as the health conditions more strongly associated with excessive sugar intake. Kidney disease was the health problem that was least associated with sugar consumption in the assessment task. However, some studies show that (added) sugar consumption is related to kidney damage [41]. 

It is noteworthy that, in contrast to the results of the free-association task, oral-health problems were rated as strongly associated with sugar intake. This discrepancy may be related to the typical separation between medical and oral healthcare systems, being the latter less prioritized [42]. For instance, the consequences of oral health problems (e.g., cavities) are possibly downplayed compared to other diseases strongly associated with excessive sugar intake (e.g., diabetes). However, this problem cannot be undervalued. For instance, tooth decay has been described as the most common non-communicable disease in Europe. In addition to all the individual consequences of this problem (e.g., impact on nutrition, self-esteem, quality of life [43,44]), this represents a great economic cost [44]. 

As in Miller et al. [28], we also observed the impact of some individual characteristics in these ratings. For instance, women considered sugar intake to be more strongly associated with several health conditions than men did. Individuals with children in the household also rated the associations with cancer and hyperactivity as stronger than those without children. Finally, experts rated the associations with obesity, cancer, and kidney disease as stronger than non-experts. Noteworthy, the association between excessive sugar intake and hyperactivity does not seem to vary according to expertise in health or nutrition, even though this association is not scientifically substantiated [27,45]. This result reveals the need to clarify the association between sugar consumption and hyperactivity, both in the general population and health professionals. Finally, we did not observe differences in ratings according to participants’ weight status (i.e., normal vs. overweight), and differences based on education level were limited to kidney disease. 

Our results must be interpreted with caution, due to the characteristics of our sample. As in other studies conducted with volunteers in the health and nutrition domain [22,46], our sample included a higher proportion of women and individuals with higher education. Another limitation of the current study concerns the rating task, which included a limited list of possible health conditions [29,30] related to excessive sugar intake. Participants may have inferred that the mere inclusion of the health conditions on the list of options signaled that they were indeed associated with excessive sugar consumption. For this reason, future studies could include a more comprehensive list of health conditions varying in their strength of association with excessive sugar intake, but also unrelated ones. 

## 5. Conclusions

An important step in developing effective interventions to reduce sugar intake is acquiring an in-depth understanding of its consumption, including the underlying individual drivers, such as consumers’ perceptions. By identifying the diseases that consumers mostly associated with excessive sugar consumption, this study can inform policymakers, educators, and health professionals. For example, interventions involving nutrition care provided by primary health professionals can promote healthy eating behaviors (for a systematic review, see [47]). Indeed, health professionals can be instrumental in sharing information and clarifying some misconceptions regarding the associations between excessive sugar intake and its adverse outcomes in health. Notably, a study with over 1500 physicians [48] showed that the majority reported counseling overweight/obese patients about the consequences of excessive SSBs. However, the most frequently addressed topic focused on the contribution of sugary beverages to weight gain, with its nutritional profile (e.g., sugar content) and a referral to an expert in nutrition being less commonly addressed. These results highlight that this approach may not be sufficient. Further studies should be directed to health professionals to understand their perceptions regarding the barriers and facilitators they face in promoting healthy eating habits in their patients. Moreover, alongside improving knowledge about the short- and long-term health consequences of excessive sugar, it is highly relevant to help individuals to identify which ingredients constitute sugar sources [33,34], how much sugar is harmful to health [28,31], and strategies to enhance comprehension and compliance with sugar-intake guidelines. It is also vital to disrupt potential optimistic bias [49] and promote individuals’ awareness of their susceptibility to sugar consumption’s health risks [50]. Finally, our results may also support the development of campaigns to raise awareness of the diverse health outcomes resulting from excessive sugar intake. As stated in an educational brochure about sugar-intake health risks (University of California—SugarScience.ucsf.edu), “Too much added sugar doesn’t just make us fat. It can also make us sick”.

## Figures and Tables

**Table 1:** Measures used to assess participants’ perceptions or behaviors toward sugar.

Measure	Item	Scale Anchors
Attention to sugar content information [34]	“How often do you look at the sugar content in the nutritional table?”	1 = *Never* to 7 = *Always*
Frequency of high sugary foods intake	“How often do you consume drinks and foods with a high sugar content?”	1 = *Never or less than once a month* to 7 = *More than once a day*
Concern about sugar intake (adapted from [35])	“I am not concerned with the amount of sugar present in my diet”.	1 = *Strongly Disagree* to 7 = *Strongly Agree* *
Health benefits from reducing sugar intake (adapted from [35])	“My health would improve if I reduced the amount of sugar in my diet”.	1 = *Strongly Disagree* to 7 = *Strongly Agree*
Intention to reduce sugar intake	“I plan to reduce the amount of sugar in my diet”.	1 = *Strongly Disagree* to 7 = *Strongly Agree*
Importance of reducing sugar intake in Portugal [33]	“In your opinion, reducing sugar consumption in the Portuguese population is …”	1 = *Not very important* to 7 = *Very important*

*Note*. * Reverse-coded.

**Table 2:** Health conditions associated with excessive sugar intake.

Health Condition	*n*	*%P*	Health Condition	*n*	*%P*
**1. Risk Factors for Cardiovascular Diseases**	**1296**		**7. Dermatologic Conditions**	**19**	
Diabetes, type II diabetes, diabetes mellitus	727	72.0	Skin, dermatological (issues), acne, pimples	16	1.6
Obesity, overweight	419	41.5	Hidradenitis suppurativa	2	0.2
Cholesterol (high), hypercholesterolemia	67	6.6	Skin inflammation	1	0.1
Hypertension, blood pressure (high)	59	5.8			
Triglycerides (high), hypertriglyceridemia	10	1.0	**8. Ophthalmic Diseases**	**17**	
Dyslipidemia	9	0.9	Blindness	4	0.4
Blood glucose	5	0.5	Glaucoma	4	0.4
			Retinopathy, diabetic retinopathy	4	0.4
**2. Cardiovascular Diseases**	**172**		Myopia	3	0.3
General (cardiovascular, cardiac, heart conditions)	116	11.5	General (eye problems)	2	0.2
Stroke	24	2.4			
Myocardial infarction, Heart attack	11	1.1	**9. Rheumatology and Osteoarticular Pathology**	**13**	
Arteriosclerosis	6	0.6	Gout, uric acid (high)	2	0.2
General (vascular conditions)	5	0.5	Osteoarticular, bones or joints (problems)	8	0.8
Circulatory problems	4	0.4	Osteoporosis	2	0.2
Thrombosis	3	0.3	Rheumatoid arthritis	1	0.1
Blood coagulation	1	0.1			
Micro and macro vasculopathy	1	0.1	**10. Urinary System Diseases**	**13**	
Ischemic fingers	1	0.1	Kidney or renal (issues, diseases), nephropathy	12	1.2
			Cystitis	1	0.1
**3. Oral Health Problems**	**76**				
Cavities	63	6.2	**11. Neurological Diseases**	**11**	
General (dental problems)	13	1.3	Neurodegenerative diseases	3	0.3
			Headaches, migraines	3	0.3
**4. Oncological Diseases**	**73**		Polyneuropathy	3	0.3
Cancer	65	6.4	Alzheimer’s disease	1	0.1
General (oncological diseases)	6	0.6	Epileptic seizures	1	0.1
Leukemia	1	0.1			
Tumors (malignant)	1	0.1	**12. General/Other Conditions**	**37**	
			Endocrine and metabolic disorders	9	0.9
**5. Mental Health Conditions**	**64**		Inflammatory processes	9	0.9
Depression, mood disorders	13	1.3	Immune system (weakened)	3	0.3
Anxiety	10	1.0	Fungal infections, candidiasis	3	0.3
Fatigue, burnout, tiredness	9	0.9	Autoimmune diseases	3	0.3
Hyperactivity	8	0.8	Respiratory system diseases	3	0.3
Stress	7	0.7	General malaise	1	0.1
Attention (deficit)	5	0.5	Aging (faster)	1	0.1
Addiction	4	0.4	Aesthetic (consequences)	1	0.1
Sleep disorders, Insomnia	2	0.2	Morbidity	1	0.1
General (mental health/psychological conditions)	2	0.2	Fluid retention	1	0.1
Memory (diminished)	2	0.2	Ulcers	1	0.1
Self-esteem (low)	1	0.1	Chronic diseases	1	0.1
Irritability	1	0.1			
					
**6. Gastrointestinal System Diseases**	**21**				
Intestine or stomach diseases	9	0.9			
Liver diseases	11	1.1			
Pancreas diseases	1	0.1			

*Note*: *n* refers to the frequency of health conditions (total = 1812). *%P* refers to the percentage of participants (N = 1010) that mentioned a given health condition.

**Table 3:** Perceived association between excessive sugar intake and health conditions, according to individual characteristics.

	Gender			Children in the Household			Expertise in Health or Nutrition ^1^		
	Men (*n* = 231)	Women (*n* = 775)	*p*	No (*n* = 672)	Yes (*n* = 338)	*p*	Non-Experts (*n* = 690)	Experts (*n* = 105)	*p*
	*M* (*SD*)	*M* (*SD*)		*M* (*SD*)	*M* (*SD*)		*M* (*SD*)	*M* (*SD*)	
Diabetes	6.70 (0.66)	6.71 (0.78)	0.876	6.70 (0.75)	6.71 (0.76)	0.810	6.69 (0.75)	6.81 (0.46)	0.024
Overweight/Obesity	6.57 (0.75)	6.72 (0.68)	0.007	6.68 (0.71)	6.70 (0.67)	0.719	6.68 (0.66)	6.81 (0.46)	0.015
Oral Health	6.57 (0.78)	6.71 (0.72)	0.016	6.66 (0.73)	6.71 (0.73)	0.270	6.70 (0.65)	6.70 (0.77)	0.896
Hyperactivity	4.72 (1.94)	5.16 (1.82)	0.001	4.93 (1.90)	5.31 (1.74)	0.002	5.10 (1.82)	5.10 (1.76)	0.962
Hypertension	4.85 (1.77)	4.89 (1.81)	0.774	4.91 (1.82)	4.84 (1.76)	0.541	4.92 (1.75)	4.90 (1.74)	0.873
Cholesterol	4.72 (1.84)	4.87 (1.76)	0.284	4.83 (1.80)	4.83 (1.73)	0.993	4.84 (1.74)	4.89 (1.70)	0.798
Cancer	4.02 (1.84)	4.87 (1.91)	<0.001	4.54 (1.95)	4.94 (1.90)	0.002	4.59 (1.91)	5.61 (1.42)	<0.001
Kidney Disease	4.35 (1.72)	4.44 (1.68)	0.465	4.38 (1.67)	4.51 (1.73)	0.244	4.41 (1.62)	4.96 (1.71)	0.001

*Note*. ^1^ Based on the description of their occupation and study area, we categorized participants according to their expertise in the health and nutrition domains. Experts include doctors, nurses, nutritionists, and pharmacists (*n* = 105; 13.2% of the total sample). The remaining participants with a higher education degree (*n* = 690) were from diverse study areas (e.g., psychology, marketing, and journalism). The *p* refers to the significance level of the difference test according to each individual characteristic (i.e., independent samples *t*-tests for gender, presence of children in the household, and expertise in health and nutrition).

**Table 4:** Descriptive results (*M* and *SD*) and correlations between the overall association ratings with the variables related to the perception and behavior toward sugar and participants’ reported lifestyle.

	*M*	*SD*	1	2	3	4	5	6	7	8	9
1. Overall association with health conditions ^a^	5.49	0.85	-								
2. Intention to reduce sugar intake	5.07	1.91	0.18 ***	-							
3. Attention to sugar information	5.11	1.86	0.21 ***	0.18 ***	-						
4. Frequency of sugar intake	3.23	1.55	−0.09 **	−0.01	−0.25 ***	-					
5. Concern about sugar intake	5.31	2.04	0.07 *	0.25 ***	0.22 ***	−0.10 **	-				
6. Health benefits from reducing sugar intake	5.23	1.98	0.16 ***	0.60 ***	0.11 **	0.09 **	0.15 ***	-			
7. Importance of reducing sugar intake in Portugal	6.73	0.69	0.20 ***	0.23 ***	0.19 **	−0.16 ***	0.21 ***	0.14 **	-		
8. Overall activity	4.27	1.54	0.03	−0.05	0.10 **	−0.12 ***	0.00	−0.13 **	0.04	-	
9. Overall health status	5.25	1.04	0.06 *	−0.04	0.17 ***	−0.19 ***	0.02	−0.16 **	0.09 *	0.40 ***	-
10. Age	36.33	13.22	−0.02	−0.05	0.02	−0.17 ***	−0.04	−0.04	0.78 *	0.00	−0.04

*Note*. *** *p* < 0.001, ** *p* < 0.010, and * *p* < 0.050. ^a^ Average of the association between excessive sugar intake and the set of eight health conditions.

## Data Availability

The data that support the findings of this study are available from the corresponding author upon reasonable request.
